# Effect of Exercise Training on Exercise Tolerance and Level of Oxidative Stress for Head and Neck Cancer Patients Following Chemotherapy

**DOI:** 10.3389/fonc.2020.01536

**Published:** 2020-08-18

**Authors:** Chia-Jui Yen, Ching-Hsia Hung, Wei-Ming Tsai, Hui-Ching Cheng, Hsin-Lun Yang, Yan-Jhen Lu, Kun-Ling Tsai

**Affiliations:** ^1^Division of Hematology and Oncology, Department of Internal Medicine, Graduate Institute of Clinical Medicine, National Cheng Kung University Hospital, Tainan, Taiwan; ^2^Department of Physical Therapy, College of Medicine, National Cheng Kung University, Tainan, Taiwan; ^3^Institute of Allied Health Sciences, College of Medicine, National Cheng Kung University, Tainan, Taiwan

**Keywords:** head and neck cancer, exercise, exercise capacity, oxidative stress, chemotherapy

## Abstract

**Background:**

Chemotherapy decreases fitness performance via repression of cardiopulmonary function and oxidative stress. This study was designed to investigate whether exercise intervention could improve exercises capacity and reduce systemic oxidative stress in patients with head and neck (H&N) cancer receiving chemotherapy.

**Methods:**

This is a single-center study. Forty-two H&N cancer patients who were undergoing chemotherapy were recruited in this study. An 8-week exercise intervention was performed by conducting the combination of aerobic and resistance exercise 3 days a week. The exercise training was conducted by a physiotherapist. The exercise capacity and exercise responses were measured from blood pressure (BP) and heart rate (HR). Oxidative stress markers from human plasma, such as total antioxidant capacity, 8-hydroxy-2′-deoxyguanosine, malondialdehyde, and carbonyl content, were tested by activity kits.

**Results:**

We provide compelling evidence that exercise training ameliorated exercise responses and increased exercise capacity by repressing resting BP and increasing 1- and 3-min BP recovery. We also found the resting HR was reduced, and the 1- and 3-min HR recovery was increased after exercise training. In addition, the rating of perceived exertion after the peak exercise was reduced after exercise intervention. We also found that exercise training repressed oxidative stress markers by elevation of total antioxidant capacity and suppression of 8-OHd and carbonyl content in plasma.

**Discussion:**

We clearly demonstrate that exercise can promote exercise capacity and reduce oxidative stress in H&N cancer patients receiving chemotherapy, which might guide new therapeutic approaches for cancer patients, especially those undergoing chemotherapy.

## Introduction

Head and neck (H&N) cancers are gaged to be one of the most prevalent cancer types in this world (1). During the past decades, chemotherapy of H&N cancer has undergone extensive testing in clinical trials, and important milestones have been achieved in several fields (2). Several chemotherapy drugs have been used in patients with H&N cancer such as cisplatin or carboplatin. Cisplatin given at a concentration of 80–120 mg/m^2^ per month causes an overall treatment response rate of 28% (3). Although chemotherapy has improved survival markedly in H&N cancer patients, a large percentage of cases with H&N severely suffered from side effects such as decreased quality of life (QoL) and physical function, neuropathy, and fatigue during treatment (4). A previous study suggested that chemotherapy not only attacks cancer cells but also influences normal tissue to cause systemic organ injury. One of the most critical problems is reduced fitness performance caused by the cardiopulmonary function during chemotherapy, which leads to low QoL (5).

Persuasive studies have concluded that cancer patients receiving chemotherapy have significant impairments in cardiorespiratory fitness and exercise capacity. The reduced physical activity suppresses cardiorespiratory fitness (6). Chemotherapy has been shown to cause muscle weakness and muscle atrophy, which reduces the diffusion transport of blood in skeletal muscle and thus results in decreased cardiorespiratory fitness (7). Moreover, deterioration of cardiac function is another important cause of reduced cardiorespiratory fitness. Chemotherapy can induce significant dysfunction in left ventricle, eventually leading to a reduction of cardiac contractility and cardiac output (8). In addition, the increased apoptosis and oxidative stress in cardiomyocytes are critical mechanisms in chemotherapy-induced cardiotoxicity (9).

A previous study reported increased formation of serum oxidative markers in patients with H&N cancer (10). In addition, chemotherapy has been reported to increase oxidative stress in cancer patients. This is obviously due to increased lipid peroxidation products that reduce free radical–scavenging capacity and antioxidant activity during chemotherapy (11). Oxidative stress has been suggested to modulate cellular signals that are required for chemotherapy to effectively destroy cancer cells (12). The systemic antioxidant capacity is suppressed, and the protein oxidation is elevated in cancer patients undergoing chemotherapy; therefore, it may be probable that oxidative stress contributes to cardiorespiratory fitness reduction in cancer subjects after cancer management (13). Antioxidants supplements can reduce or prevent side effects caused by chemotherapy, but they may diminish the clinical effectiveness of chemotherapeutic treatments (14). Thus, to find effective interventions rather than antioxidants supplements for cancer patients is important.

Exercise is very helpful for the recovery from cancer treatments. Moderate-intensity exercise training can improve cancer patients’ mental health as compared to non-exercise subjects (15). In addition, a previous study has also suggested that appropriate exercise can improve the discomforts caused by chemotherapy in cancer patients. Mohamady et al. (16) reported that 12 weeks of moderate-intensity aerobic exercise enriched hemoglobin and erythrocytes in the circulatory system, thereby improving the clinical symptoms of anemia. However, whether exercise training can reduce oxidative stress in H&N cancer patients receiving chemotherapy is still unclear. The primary aim of this present study was to investigate the effect of 8 weeks’ combinative exercise training on cardiorespiratory fitness in patients receiving chemotherapy. We hypothesized that exercise intervention ameliorates exercise responses and exercise capacity in cancer subjects undergoing chemotherapy. The secondary aim was to examine changes in oxidative stress marker in patient plasma. The hypothesis was that exercise training reduces oxidative stress markers in cancer subjects undergoing chemotherapy.

## Materials and Methods

This single-center study was conducted between September 2016 and December 2017 as a single-arm study. The National Cheng Kung University and National Cheng Kung University Hospital were responsible for the integrity and conduct of this study. This study was approved by the ethical committee of National Cheng Kung University Hospital Institutional Review Board, Tainan, Taiwan (B-ER-105-102), and this trial was registered in Thai Clinical Trials Registry (TCTR20171211001). After explaining all the experimental procedures in detail, each case provided written consent to join in the study.

### Subjects

Head and neck cancer patients were recruited from the Department of Hematology and Oncology at the National Cheng Kung University Hospital, Tainan, Taiwan. The patient sample size was referred by previous cancer exercise reports (17, 18). The characteristics of the studied population are presented in Table 1. The inclusion criteria included (1) age 20 years or older with H&N cancer; (2) H&N cancer diagnosed by pathology, cytology, or imaging; (3) no serious complications; (4) no brain tumor metastasis; and (5) no history of mental illness. The exclusion criteria included (1) those who could not sign the consent, (2) neurological disorders (e.g., stroke), (3) pregnant or lactating women, (4) severe psychiatric disorders (e.g., bipolar disorder and schizophrenia), (5) musculoskeletal disorders that limited mobility (e.g., myopathy, amputation), (6) severe organ failure, and (7) clinically determined to have a survival rate of less than 3 months.

**TABLE 1:** Basic characteristics of subjects.

Basic characteristics	Exercise group (*N* = 30)
Gender	
Female	7
Male	23
Risk factor	
Smoke	15
Drink	20
Chew the betel nuts	13
Age (years)	56 ± 12.3
Height (m)	1.6 ± 0.7
Body weight (kg)	64.6 ± 11.2
Chemotherapy drugs	
Cisplatin	22
Gemcitabine	1
Medroxyprogesterone	7

### Exercise Intervention

The subjects undergoing chemotherapy were arranged to receive 8 weeks of exercise training. Aerobic exercise and resistance exercise were included in the exercise program for patients. The participants were asked to perform exercise training 3 days per week in the gym in the National Cheng Kung University supervised by a physiotherapist. The training intensity was based on the American College of Sports Medicine’s cancer patient guidelines: using moderate-intensity exercise training, with the intensity of the maximum heart rate (HR) ranging between 60 and 70%, where the maximum HR calculation formula is usually 220 − age (19). Forty to fifty minutes of training time included a 5-min warm-up, aerobic exercise training for 30 min, and a 5-min cool-down. In addition, the TheraBand resistance band was used for resistance exercise in this study. Resistance exercise was performed by TheraBand at the intensity of the rating of perceived exertion (RPE) scale from “somewhat heavy” to “heavy.” Each exercise consists of 10 to 12 repetitions for one set, three sets per training. Both upper and lower extremities were trained.

### Primary Outcome Measure–Exercise Responses

The cardiovascular physiological parameters, including blood pressure (BP), HR, SpO_2_, and RPE, were measured in this study. Before and after exercise training, subjects were asked to perform an exercise to test the maximal exercise capacity and exercise responses. From the first day of recruitment to the last day of exercise training, the parameters in 1 and 3 min of recovery time were recorded after exercise testing.

### Secondary Outcome Measures–Measurement of Oxidative Stress

Human plasma was isolated from total blood collected from the first day of recruitment to the last day of exercise training. Total antioxidant capacity, 8-hydroxy-2′-deoxyguanosine (8-OHdG), and carbonyl levels were measured using commercial kits (ab65329, ab201734, and ab126287; Abcam) according to the manufacturer’s instructions. The malondialdehyde (MDA) level was determined by a commercial kit (MAK085, Sigma) according to the manufacturer’s instructions.

## Statistics

Data were expressed as mean ± standard deviations for all variables. No adjustment was made for analysis. The paired *t*-test was used to measure the differences in the mean values of the parameters between preintervention and postintervention. Statistical significance was set at *p* < 0.05. All analyses were done using SPSS version 22.0 (SPSS Inc., Chicago, IL, United States).

## Results

### Participant Flow and Recruitment

**FIGURE 1:**
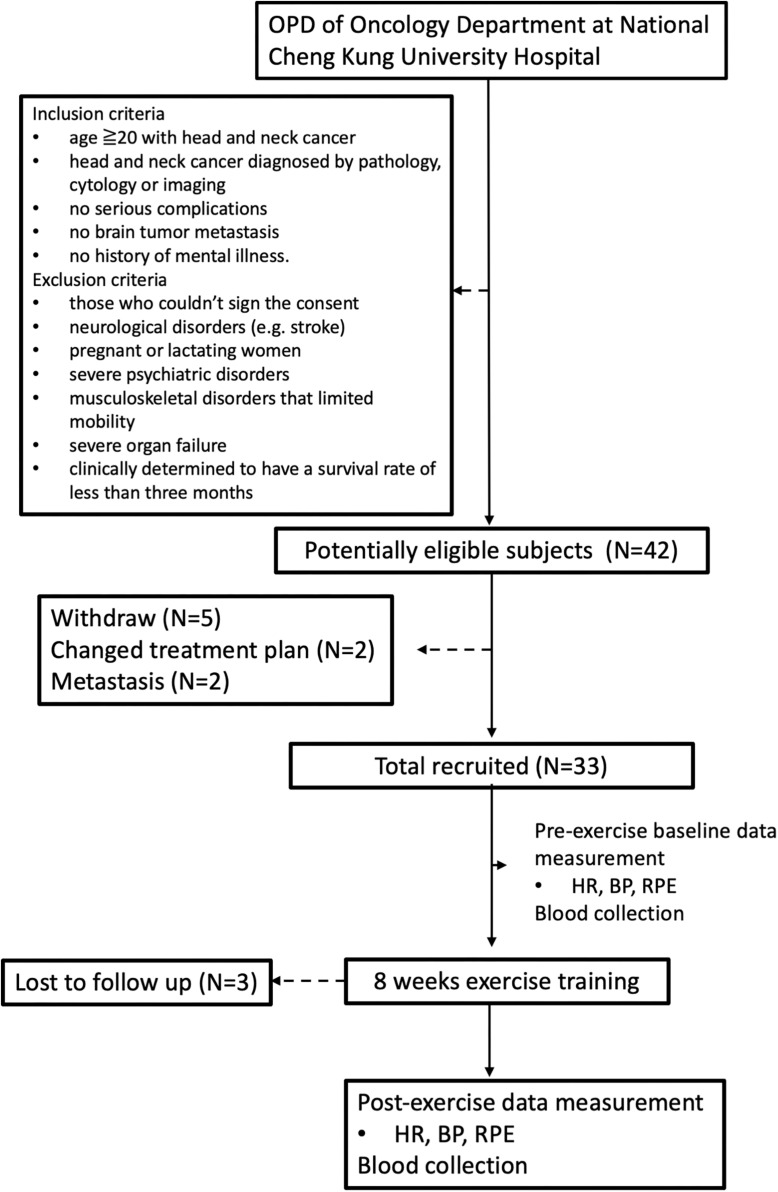
Flow of participants through the trial.

A total of 42 patients were screened in this study, 30 subjects were enrolled, and 12 subjects were excluded including 5 subjects who withdrew from participation, 2 subjects with changed treatment plan, 2 subjects with severe metastasis, and 3 subjects lost to follow-up (Figure 1). All participants completed 8 weeks’ exercise training and data collection.

### Demographic Data

Participant demographics are shown in Table 1. A total of 23 of 30 participants were male; a total of 7 of 30 participants were female. The mean ± SD age of the total subjects was 56 ± 12.3 years. The mean ± SD height of the total subjects was 1.6 ± 0.7 m, and the mean body weight of the total subjects was 64.4 ± 11.2 kg. All participants had undergone chemotherapy (22 subjects were treated with cisplatin, 1 case was treated with gemcitabine, 7 subjects were treated with medroxyprogesterone).

### Outcomes

#### Blood Pressure

As shown in Table 2, the results from BP revealed that resting systolic BP (SBP) and dystolic BP (DBP) are lower after exercise training (SBP pretraining 111 ± 18.7, SBP posttraining 106.8 ± 12.1; DBP pretraining 69.6 ± 12.6, SBP posttraining 64 ± 10.6; all *p* < 0.05). There was no significant difference in both SBP and DBP in peak exercise before and after exercise training. In addition, exercise training increased 1- and 3-min BP recovery in DBP (*p* < 0.05) and increased 3-min BP recovery in SBP (*p* < 0.05) (Table 2).

**TABLE 2:** Blood pressure, heart rate, oxygen saturation, and rate of perceived exertion at pretraining/posttraining.

	Subjects (*N* = 30)	
	Pretraining	Posttraining
**Blood pressure**		
Rest		
SBP (mmHg)	111 ± 18.7	106.8 ± 12.1*
DBP (mmHg)	69.6 ± 12.6	64 ± 10.6*
Peak		
SBP (mmHg)	156.9 ± 24.3	157.6 ± 22.7
DBP (mmHg)	82.7 ± 16.4	83.7 ± 13.8
1-min Recovery		
SBP (mmHg)	111.8 ± 22	108.9 ± 13.2
DBP (mmHg)	70.2 ± 14.6	66.4 ± 11.3*
3-min Recovery		
SBP (mmHg)	105.1 ± 15.9	100.2 ± 11.9*
DBP (mmHg)	66.8 ± 12.9	60.9 ± 10.6*
**Heart rate**		
Rest HR (beats)	85.2 ± 12.1	76.4 ± 10.2*
Peak HR (beats)	131 ± 15.8	132.5 ± 14
Rest 1-min HR (beats)	99.3 ± 13.3	93.6 ± 12.1*
Rest 3-min HR (beats)	94.8 ± 12.1	85 ± 13.2*
Rest 1-min HRR (beats)	31.6 ± 14.6	38.9 ± 15.2*
Rest 3-min HRR (beats)	36.2 ± 14.6	47.5 ± 16.4*
**Oxygen saturation**		
Rest SpO_2_	97.8 ± 1.3	97.9 ± 1.1
Peak SpO_2_	97 ± 1.5	97.1 ± 2.5
Rest 1-min SpO_2_	97.2 ± 1.4	97.4 ± 1.4
Rest 3-min SpO_2_	97 ± 1.6	97.4 ± 1.7
**Rate of perceived exertion**		
Rest RPE	10.8 ± 0.4	10.8 ± 0.4
Peak RPE	13.4 ± 1.3	13.2 ± 1.1
Rest 1-min RPE	11.2 ± 0.7	10.9 ± 0.5*
Rest 3-min RPE	10.8 ± 0.7	10.8 ± 20.5

*p < 0.05 pretraining versus posttraining. SBP, systolic blood pressure; DBP, diastolic blood pressure; HR, heart rate; HRR, heart rate recovery; SpO_2_, oxygen saturation; RPE, rate of perceived exertion.

#### Heart Rate

The results from HR indicated that resting HR is lower after exercise training (rest HR pretraining 85.2 ± 12.1, rest HR posttraining 76.4 ± 10.2; *p* < 0.05). There was no significant difference in HR in peak exercise before and after exercise training. Moreover, exercise training reduced 1- and 3-min HR after peak exercise (1-min HR pretraining 99.3 ± 13.3, 1-min HR posttraining 93.6 ± 12.1; 3-min HR pretraining 94.8 ± 12.1, 3-min HR posttraining 85 ± 13.2; all *p* < 0.05). A similar result had been found in HR recovery (HRR) data (all *p* < 0.05).

#### Oxygen Saturation

There was no significant difference in oxygen saturation in resting or peak exercise before and after exercise training.

#### Rate of Perceived Exertion

There was no significant difference in RPE in resting or peak exercise before and after exercise training. However, exercise training reduced 1-min RPE after peak exercise (1-min RPE pretraining 11.2 ± 0.7, 1-min RPE posttraining 10.9 ± 0.5; *p* < 0.05).

#### Oxidative Stress Markers

We found that exercise training significantly increased total antioxidant capacity (pretraining 221.7 ± 62.2, posttraining 443.7 ± 72.1; *p* < 0.05). Moreover, plasma levels of carbonyl content and 8-OHdG were decreased after exercise training (carbonyl content pretraining 10.1 ± 2.6, carbonyl content posttraining 5.5 ± 1.8; 8-OHdG pretraining 1031.3 ± 43.8, 8-OHdG posttraining 761.3 ± 66.3; all *p* < 0.05). However, level of MDA was not changed in plasma (Table 3).

**TABLE 3:** The oxidative stress markers at pretraining/posttraining.

	Subjects (*N* = 30)	
	Pretraining	Posttraining
Total antioxidant capacity (μM)	221.7 ± 62.2	443.7 ± 72.1*
8-OHdG (ng/mL)	1031.3 ± 43.8	761.3 ± 66.3*
MDA (nmol/mL)	4.7 ± 0.8	3.8 ± 1.3
Carbonyl content (nmol/mg protein)	10.1 ± 2.6	5.5 ± 1.8*

*p < 0.05 pretraining versus posttraining. 8-OHdG, 8-hydroxy-2′-deoxyguanosine; MDA, malondialdehyde.

## Discussion

This is the first study to explore the effect of exercise training on cardiovascular physiological parameters and oxidative stress markers in H&N cancer patients receiving chemotherapy. Here, we clearly demonstrated that exercise training ameliorated exercise responses by repression of resting HR and BP and promotion of HR and BP recovery. We also proved that exercise training mitigated decreased systemic oxidative stress in H&N cancer patients receiving chemotherapy.

Chemotherapy drugs are extensively used to remedy patients with H&N cancer. A previous report revealed that chemotherapy drugs not only attack cancer cells but also influence normal tissues to cause systemic organ injury. One of the most important issues is decreased QoL and reduced fitness performance resulting from repression of cardiopulmonary function (5). In addition, increased systemic oxidative stress is also considered to be a side effect of chemotherapy (20). In the present study, we demonstrated that exercise training not only improved exercise responses and exercise capacity but also mitigated systemic oxidative stress in H&N cancer patients receiving chemotherapy.

High BP has been positively associated with the mortality rate in various cardiovascular diseases (21). Hypertension is one of the clinical side effects after chemotherapy treatments. Chemotherapy drugs impair vascular endothelial function and repressed nitric oxide (NO) synthesis, thereby causing vasoconstriction (22). In addition, a clinical trial reported that 144 cases who received chemotherapy developed elevated BP, with an incidence rate ranging from 8 to 67% (23). Notably, exercise training can effectively mitigate the incidence of cardiovascular disease by 30% and the incidence of mortality by 52% (24). Body vascular resistance can be reduced after exercise (25), which may result from that exercise decreases the levels of reactive blood oxygen species and thus lowers BP (26). In the present study, we found that exercise intervention reduced resting SBP and DBP and improved 1-min DBP recovery, 3-min SBP recovery, and 3-min DBP recovery, suggesting that exercise intervention has potential to ameliorate exercise responses in H&N cancer patients receiving chemotherapy (Table 2). Results from this part indicated that exercise training ameliorates vessel adaptation in H&N cancer patients receiving chemotherapy.

The HR response to exercise is reported to a complex interaction among many factors such as age, physical conditioning, and sympathetic system, as well as venous return (27). Previous investigation has reported a correlation between non-success to achieve a predicted HR and the prognosis of cardiovascular disease (28). Jones et al. (29) indicated that chemotherapy medications have deleterious influences on the autonomic nervous system and motive endocrine system alteration that in turn affect the cardiovascular function and sympathetic and parasympathetic interplays. Exercise intervention had been suggested to repress the activity of the sympathetic nervous system and promote vasodilatation, thereby reducing the resting HR (30). Exercise training increases exercise capacity with the elevated peak HR; in addition, previous studies have found that long-term endurance exercise training can reduce the β1-adrenergic receptor in the right atrium because the β1-adrenergic receptor can receive sympathetic information, whereas aerobic endurance training increases cardiac parasympathetic activity that suppresses sympathetic activity and leads to declines in HR (31–33). In our study, we confirmed that exercise training decreased resting HR and increased 1-min HR and 3-min HRR in H&N cancer patients receiving chemotherapy. Results from this part indicated that exercise training mends HR responses in H&N cancer patients receiving chemotherapy, which might be through ameliorating the balance of sympathetic and parasympathetic interplays.

The chemotherapy-induced cardiotoxicity has been connected to several mechanisms, including elevated production of reactive oxygen species, increased lipid peroxidation, mitochondrial impairment, and elevation of Ca^2++^ concentration leading to systemic oxidative stress (34). Besides, chemotherapy drugs have been suggested to be involved in the elevation of nitrosative stress by up-regulated formation of potent reactive nitrogen species, such as peroxynitrite, by the action of superoxide with NO (35). Expression of cardiac NO was found to be induced during the progression of chemotherapy-facilitated cardiomyopathy by increased expression level of inducible NO synthase (36). Elevated oxidative stress acts as a facilitator for the progression of inflammation, leading to dysfunction of cardiovascular system (36). Previous reports suggested that exercise is a useful intervention to prevent several risk factors of cardiovascular disease. For example, exercise intervention furnishes various positive effects on human endothelial function and maintains cardiovascular homeostasis by activation of antioxidative capacities (37). Our data show that exercise training increased total antioxidant capacity and reduced 8-OHdG and carbonyl content levels in H&N cancer patients receiving chemotherapy, indicating that exercise may protect against cardiovascular dysfunction caused by chemotherapy repressing systemic oxidative stress.

Most approaches for cancer case involve chemotherapy that induces injuries to the patient’s body. In addition, the systemic side effects may affect their daily life activities and reduce their QoL and exercise capacity. In our study, exercise training was shown to have beneficial effects on cardiovascular physiological responses exercise capacity in H&N cancer cases receiving chemotherapy.

## Conclusion

In summary, chemotherapy not only attacks cancer tissues but also influences normal tissues to cause systemic damages. One of the most critical causes in QoL repression is reduced fitness performance resulting from the impairment of cardiopulmonary function and exercise capacity. In addition, high oxidative stress has been involved in cardiotoxicity caused by chemotherapy. In this study, we clearly demonstrated that exercise training ameliorated exercise responses and increased exercise capacity in H&N cancer patients receiving chemotherapy, which may be attributed to decreased systemic oxidative stress. Our data provide a well-distinguished pathway to guide new therapeutic approaches for treating patients receiving chemotherapy.

## Data Availability Statement

The raw data supporting the conclusions of this article will be made available by the authors, without undue reservation, to any qualified researcher.

## Ethics Statement

The studies involving human participants were reviewed and approved by the National Cheng Kung University. The patients/participants provided their written informed consent to participate in this study.

## Author Contributions

C-JY, C-HH, W-MT, and K-LT participated in study design. H-CC participated in acquisition of data. H-LY, Y-JL, and K-LT participated in interpretation of data. C-JY, C-HH, and K-LT helped to draft the manuscript. C-HH and K-LT contributed to the conception and design of the study and final approval of the submitted version. All authors read and approved the final manuscript.

## Conflict of Interest

The authors declare that the research was conducted in the absence of any commercial or financial relationships that could be construed as a potential conflict of interest.

## References

[1] Iglesias DLC Arrazubi AV Baste RN Carral MA Cirauqui CB Escobar Y SEOM clinical guidelines for the treatment of head and neck cancer (2017). *Clin Transl Oncol.* (2018) 20:75–83. 10.1007/s12094-017-1776-1 29159792 PMC5785598

[2] Dimery IW Hong WK. Overview of combined modality therapies for head and neck cancer. *J Natl Cancer Inst.* (1993) 85:95–111. 10.1093/jnci/85.2.95 8418313

[3] Veronesi A Zagonel V Tirelli U Galligioni E Tumolo S Barzan L High-dose versus low-dose cisplatin in advanced head and neck squamous carcinoma: a randomized study. *J Clin Oncol.* (1985) 3:1105–8. 10.1200/jco.1985.3.8.1105 4040550

[4] Kalter J Kampshoff CS Chinapaw MJ Vanm W Galindo-Garre F Schep G Mediators of exercise effects on hrqol in cancer survivors after chemotherapy. *Med Sci Sports Exerc.* (2016) 48:1859–65. 10.1249/mss.0000000000000976 27128668

[5] Wall BA Galvao DA Fatehee N Taaffe DR Spry N Joseph D Maximal exercise testing of men with prostate cancer being treated with androgen deprivation therapy. *Med Sci Sports Exerc.* (2014) 46:2210–5. 10.1249/mss.0000000000000353 24694745

[6] Loewen GM Watson D Kohman L Herndon JE II Shennib H Kernstine K Preoperative exercise Vo2 measurement for lung resection candidates: results of Cancer and Leukemia Group B Protocol 9238. *J Thorac Oncol.* (2007) 2:619–25. 10.1097/jto.0b013e318074bba7 17607117

[7] Galvao DA Spry NA Taaffe DR Newton RU Stanley J Shannon T Changes in muscle, fat and bone mass after 36 weeks of maximal androgen blockade for prostate cancer. *BJU Int.* (2008) 102:44–7. 18336606 10.1111/j.1464-410X.2008.07539.x

[8] Swain SM Whaley FS Ewer MS. Congestive heart failure in patients treated with doxorubicin: a retrospective analysis of three trials. *Cancer.* (2003) 97:2869–79. 10.1002/cncr.11407 12767102

[9] Octavia Y Tocchetti CG Gabrielson KL Janssens S Crijns HJ Moens AL. Doxorubicin-induced cardiomyopathy: from molecular mechanisms to therapeutic strategies. *J Mol Cell Cardiol.* (2012) 52:1213–25. 10.1016/j.yjmcc.2012.03.006 22465037

[10] Singh AK Pandey P Tewari M Pandey HP Gambhir IS Shukla HS. Free radicals hasten head and neck cancer risk: a study of total oxidant, total antioxidant, DNA damage, and histological grade. *J Postgrad Med.* (2016) 62:96–101. 10.4103/0022-3859.180555 27089108 PMC4944358

[11] Subramaniam S Subramaniam S Shyamala Devi CS. Erythrocyte antioxidant enzyme activity in CMF treated breast cancer patients. *Cancer Biochem Biophys.* (1994) 14:177–82. 7728738

[12] Shacter E Williams JA Hinson RM Senturker S Lee YJ. Oxidative stress interferes with cancer chemotherapy: inhibition of lymphoma cell apoptosis and phagocytosis. *Blood.* (2000) 96:307–13. 10.1182/blood.v96.1.307.013k47_307_313 10891466

[13] Repka CP Hayward R. Oxidative stress and fitness changes in cancer patients after exercise training. *Med Sci Sports Exerc.* (2016) 48:607–14. 10.1249/mss.0000000000000821 26587845 PMC4979000

[14] Lee YJ Shacter E. Oxidative stress inhibits apoptosis in human lymphoma cells. *J Biol Chem.* (1999) 274:19792–8. 10.1074/jbc.274.28.19792 10391922

[15] Lesiuk T. The effect of mindfulness-based music therapy on attention and mood in women receiving adjuvant chemotherapy for breast cancer: a pilot study. *Oncol Nurs Forum.* (2015) 42:276–82. 10.1188/15.onf.276-282 25901379

[16] Mohamady HM Elsisi HF Aneis YM. Impact of moderate intensity aerobic exercise on chemotherapy-induced anemia in elderly women with breast cancer: a randomized controlled clinical trial. *J Adv Res.* (2017) 8:7–12. 10.1016/j.jare.2016.10.005 27872759 PMC5109847

[17] Gehring K Kloek CJ Aaronson NK Janssen KW Jones LW Sitskoorn MM Feasibility of a home-based exercise intervention with remote guidance for patients with stable grade II and III gliomas: a pilot randomized controlled trial. *Clin Rehabil.* (2018) 32:352–66. 10.1177/0269215517728326 28882061 PMC6625754

[18] Kim K Gu MO Jung JH Hahm JR Kim SK Kim JH Efficacy of a home-based exercise program after thyroidectomy for thyroid cancer patients. *Thyroid.* (2018) 28:236–45. 10.1089/thy.2017.0277 29258382

[19] Garber CE Blissmer B Deschenes MR Franklin BA Lamonte MJ Lee IM American College of Sports Medicine position stand. Quantity and quality of exercise for developing and maintaining cardiorespiratory, musculoskeletal, and neuromotor fitness in apparently healthy adults: guidance for prescribing exercise. *Med Sci Sports Exerc.* (2011) 43:1334–59. 10.1249/mss.0b013e318213fefb 21694556

[20] Yang Z Schumaker LM Egorin MJ Zuhowski EG Guo Z Cullen KJ. Cisplatin preferentially binds mitochondrial DNA and voltage-dependent anion channel protein in the mitochondrial membrane of head and neck squamous cell carcinoma: possible role in apoptosis. *Clin Cancer Res.* (2006) 12:5817–25. 10.1158/1078-0432.ccr-06-1037 17020989

[21] Muntner P Krousel-Wood M Hyre AD Stanley E Cushman WC Cutler JA Antihypertensive prescriptions for newly treated patients before and after the main antihypertensive and lipid-lowering treatment to prevent heart attack trial results and seventh report of the joint national committee on prevention, detection, evaluation, and treatment of high blood pressure guidelines. *Hypertension.* (2009) 53:617–23. 10.1161/hypertensionaha.108.120154 19221214

[22] Tsai KL Huang PH Kao CL Leu HB Cheng YH Liao YW Aspirin attenuates vinorelbine-induced endothelial inflammation via modulating SIRT1/AMPK axis. *Biochem Pharmacol.* (2014) 88:189–200. 10.1016/j.bcp.2013.12.005 24345330

[23] Gressett SM Shah SR. Intricacies of bevacizumab-induced toxicities and their management. *Ann Pharmacother.* (2009) 43:490–501. 10.1345/aph.1l426 19261963

[24] Sakamoto S Yokoyama N Tamori Y Akutsu K Hashimoto H Takeshita S. Patients with peripheral artery disease who complete 12-week supervised exercise training program show reduced cardiovascular mortality and morbidity. *Circulat J.* (2009) 73:167–73. 10.1253/circj.cj-08-0141 19039192

[25] Hegde SM Solomon SD. Influence of physical activity on hypertension and cardiac structure and function. *Curr Hypert Rep.* (2015) 17:77. 10.1007/s11906-015-0588-3 PMC4624627 26277725

[26] Haack KK Zucker IH. Central mechanisms for exercise training-induced reduction in sympatho-excitation in chronic heart failure. *Autonom Neurosci.* (2015) 188:44–50. 10.1016/j.autneu.2014.10.015 25458427 PMC4336572

[27] Hammond HK Froelicher VF. Normal and abnormal heart rate responses to exercise. *Prog Cardiovasc Dis.* (1985) 27:271–96. 10.1016/0033-0620(85)90010-6 2857054

[28] Ellestad MH Wan MK. Predictive implications of stress testing. Follow-up of 2700 subjects after maximum treadmill stress testing. *Circulation.* (1975) 51:363–9. 10.1161/01.cir.51.2.363 1112017

[29] Jones LW Courneya KS Mackey JR Muss HB Pituskin EN Scott JM Cardiopulmonary function and age-related decline across the breast cancer survivorship continuum. *J Clin Oncol.* (2012) 30:2530–7. 10.1200/jco.2011.39.9014 22614980 PMC3397786

[30] Kingwell BA. Nitric oxide as a metabolic regulator during exercise: effects of training in health and disease. *Clin Exp Pharmacol Physiol.* (2000) 27:239–50. 10.1046/j.1440-1681.2000.03232.x 10779120

[31] Hammond HK White FC Brunton LL Longhurst JC. Association of decreased myocardial beta-receptors and chronotropic response to isoproterenol and exercise in pigs following chronic dynamic exercise. *Circulat Res.* (1987) 60:720–6. 10.1161/01.res.60.5.720 3036395

[32] Carter JB Banister EW Blaber AP. Effect of endurance exercise on autonomic control of heart rate. *Sports Med (Auckland NZ).* (2003) 33:33–46. 10.2165/00007256-200333010-00003 12477376

[33] Angadi SS Jarrett CL Sherif M Gaesser GA Mookadam F. The effect of exercise training on biventricular myocardial strain in heart failure with preserved ejection fraction. *ESC Heart Fail.* (2017) 4:356–9. 10.1002/ehf2.12149 28772048 PMC5542728

[34] Ludke AR Al-Shudiefat AA Dhingra S Jassal DS Singal PK. A concise description of cardioprotective strategies in doxorubicin-induced cardiotoxicity. *Can J Physiol Pharmacol.* (2009) 87:756–63. 19898559 10.1139/Y09-059

[35] Mukhopadhyay P Rajesh M Batkai S Kashiwaya Y Hasko G Liaudet L Role of superoxide, nitric oxide, and peroxynitrite in doxorubicin-induced cell death in vivo and in vitro. *Am J Physiol Heart Circ Physiol.* (2009) 296:H1466–83. 19286953 10.1152/ajpheart.00795.2008 PMC2685360

[36] Singh S Gupta AK. Nitric oxide: role in tumour biology and iNOS/NO-based anticancer therapies. *Cancer Chemother Pharmacol.* (2011) 67:1211–24. 10.1007/s00280-011-1654-4 21544630

[37] Park Y Booth FW Lee S Laye MJ Zhang C. Physical activity opposes coronary vascular dysfunction induced during high fat feeding in mice. *J Physiol.* (2012) 590:4255–68. 10.1113/jphysiol.2012.234856 22674721 PMC3473283

